# Iron supplementation enhances RSL3-induced ferroptosis to treat naïve and prevent castration-resistant prostate cancer

**DOI:** 10.1038/s41420-023-01383-4

**Published:** 2023-03-06

**Authors:** Federica Maccarinelli, Daniela Coltrini, Silvia Mussi, Mattia Bugatti, Marta Turati, Paola Chiodelli, Arianna Giacomini, Floriana De Cillis, Nadia Cattane, Annamaria Cattaneo, Alessia Ligresti, Michela Asperti, Maura Poli, William Vermi, Marco Presta, Roberto Ronca

**Affiliations:** 1 University of Brescia, Department of Molecular and Translational Medicine, Brescia, Italy; 2 Biological Psychiatry Unit, IRCCS Istituto Centro San Giovanni di Dio Fatebenefratelli, Brescia, Italy; 3 Institute of Biomolecular Chemistry, National Research Council of Italy, Pozzuoli, Italy

**Keywords:** Prostate cancer, Chemotherapy

## Abstract

Prostate cancer (PCa) is a leading cause of death in the male population commonly treated with androgen deprivation therapy that often relapses as androgen-independent and aggressive castration-resistant prostate cancer (CRPC). Ferroptosis is a recently described form of cell death that requires abundant cytosolic labile iron to promote membrane lipid peroxidation and which can be induced by agents that inhibit the glutathione peroxidase-4 activity such as RSL3. Exploiting in vitro and in vivo human and murine PCa models and the multistage transgenic TRAMP model of PCa we show that RSL3 induces ferroptosis in PCa cells and demonstrate for the first time that iron supplementation significantly increases the effect of RSL3 triggering lipid peroxidation, enhanced intracellular stress and leading to cancer cell death. Moreover, the combination with the second generation anti-androgen drug enzalutamide potentiates the effect of the RSL3 + iron combination leading to superior inhibition of PCa and preventing the onset of CRPC in the TRAMP mouse model. These data open new perspectives in the use of pro-ferroptotic approaches alone or in combination with enzalutamide for the treatment of PCa.

## Introduction

Prostate cancer (PCa) represents the third most common malignancy worldwide and is a leading cause of death in the male population [1]. In the clinic, prostate lesions are extremely heterogeneous and characterized by different clinical behaviors ranging from premalignant to very aggressive lethal tumors [2]. To date, the standard care for PCa patients is represented by the androgen deprivation therapy (ADT) [3, 4]. In most cases, after an initial and successful response of about 12–24 months, prostate tumors regrow and become independent from androgens originating the so called stage termed castration-resistance prostate cancer (CRPC), that is characterized by poor prognosis [5].

Iron (Fe) is an essential redox element directly involved in many pivotal cell functions ranging from oxygen transport through hemoglobin to cell detoxification. In pathological conditions like cancer, iron has been found to be closely related to the key processes regulating tumor onset and progression [6, 7]. Indeed, tumor cells are extremely stingy with iron that is involved and required in various biochemical pathways during tumor progression [8]. Moreover, it has been reported that disorders of iron metabolism can facilitate tumor growth, angiogenesis and metastasis [6, 9, 10]. Despite its crucial role in cell growth, this transition metal represents a potentially toxic element since iron-catalyzed reactive oxygen species (ROS) may form if its storage and redox status are not properly controlled [11]. In fact, the deregulation of intracellular iron metabolism may represent a damaging entity for tumor cells, and various studies have reported the pivotal role of ferroptosis, an iron-dependent form of cell death, in killing cancer cells and suppressing tumor growth [12, 13].

Ferroptosis has been described as a non-apoptotic and non-necrotic oxidative form of cell death dependent from the availability of abundant cytosolic labile iron that triggers membrane lipid peroxidation [14, 15] and cancer cells have been shown to be particularly sensitive to ferroptosis [6, 7, 16]. Ferroptosis is defined by a series of specific phenotypical, molecular and biochemical features that include cellular and subcellular morphological changes, and the production of ferroptosis-related molecules (such as labile iron, ROS, peroxidized lipids and GSH) [17]. The major pathways involved in ferroptosis include the Xc- cystine uptake system, the glutathione peroxidase-4 (GPX4) activity, and the polyunsaturated/monounsaturated fatty acids (PUFAs/MUFAs) biosynthesis. The study of ferroptosis in vitro and in vivo is essentialy based on the use of specific inducers that modulate the GPX4 activity like Erastin, that indirectly inhibits GPX4 by binding the cystine-glutamate antiporter Xc- at the plasma membrane leading to glutathione depletion, or RSL3 that directly binds and inhibits GPX4 [14]. On the other hand, specific inhibitors of lipid peroxidation like Ferrostatin-1 or iron chelators are widely used to better characterize ferroptotic death [18]. To date, most of the research on ferroptosis in cancer biology and therapy has been carried out in vitro or in immune-deficient xenograft models, where the key features and interections of the native microenvironment are obviously lost. For this reason, integrated studies covering both human and murine immunocompentent models are eagerly required.

In this work we exploit an “integrated platform” represented by human and murine PCa cells and the in vivo multistage TRAMP mouse model [19, 20] to better define the therapeutic potential of ferroptosis in naïve and androgen independent settings [21]. We show that RSL3 efficiently induces ferroptosis in human and murine PCa cells and demonstrate for the first time that iron supplementation potentiates RSL3-induced ferroptosis in PCa cells in vitro, in vivo, and in the multistage TRAMP model of PCa. In addition, the combination with the second generation anti-androgen receptor enzalutamide increases the potency of the iron+RSL3 combination resulting in a superior blockade of tumor growth.

## Results

### Iron supplementation enhances ferroptotic cell death in PCa cells in vitro

Iron overload [21] and ferroptosis inducers [22] have been shown to induce cell death in PCa cells. Here, we evaluated the possibility to potentiate the ferroptotic cell death in PCa cells exploiting the combination of the ferroptosis inducer RSL3 and iron supplementation obtained in vitro by treatment with ferric ammonium citrate (FAC).

**Fig. 1:**
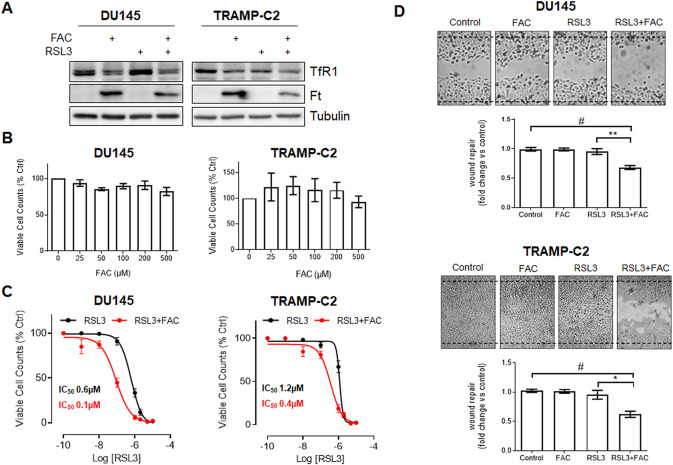
In vitro effect of RSL3+FAC treatment on PCa cells. **A** Western blot analysis of transferrin receptor (TfR-1) and Ferritin (Ft) in DU145 and TRAMP-C2 cells treated for 24 h with 100 μM FAC, 0.5 μM RSL3 or RSL3 + FAC. Tubulin was used for normalization. Viable cell count of DU145 and TRAMP-C2 cells treated for 48 h with increasing concentrations of FAC (25–500 μM) (**B**), RSL3 (0.0001–10 μM) or RSL3 + FAC (0.0001–10 μM RSL3 + 100 μM FAC) (**C**). **D** Motility assay performed with DU145 and TRAMP-C2 treated for 24 h with 100 μM FAC and RSL3 (0.3 μM for DU-145 cells and 1.0 μM for TRAMP-C2 cells). Representative images are reported. Data are the mean ± SEM of at least 3 experiments (*p < 0.05, **p < 0.01, ***p < 0.001, ^*#*^*p* < 0.0001).

In basal conditions, both human DU145 and murine TRAMP-C2 PCa cell lines express the main cellular iron importer transferrin receptor-1 (TfR1). As shown in Fig. 1A, TfR1 expression is decreased by treatment with FAC, while levels of the major intracellular iron-storage protein ferritin (Ft), are significantly increased indicating that iron was taken up by tumor cells.

**Fig. 2:**
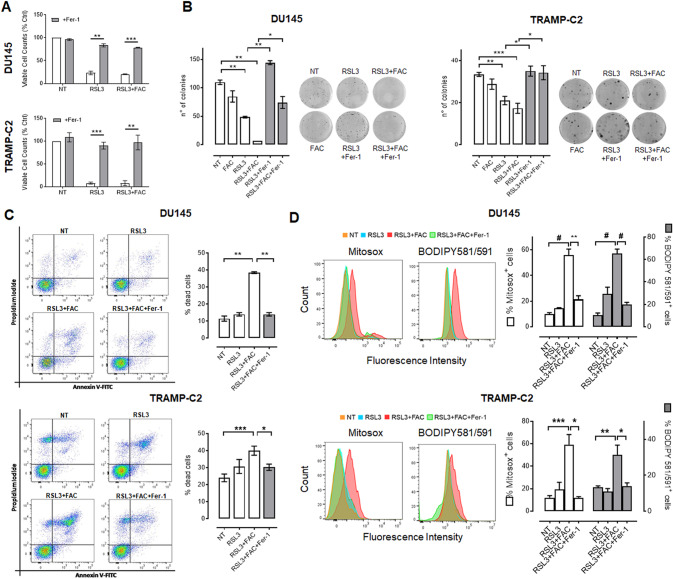
RSL3+FAC treatment induces ferroptosis in PCa cells. **A** Viable cell counts of DU145 and TRAMP-C2 cells treated for 48 h with RSL3 (1 μM for DU-145 cells and 2 μM for TRAMPC2 cells) or RSL3 + FAC (0.3 μM for DU-145 cells and 1 μM for TRAMPC2 cells + 100 μM FAC) in the presence or not of the selective inhibitor of the ferroptosis process Ferrostatin-1 (10 μM Fer-1). **B** Quantification and representative images of colony formation assay after 24 h treatment with 1 μM FAC and RSL3 (0.1 μM for DU-145 cells and 0.5 μM for TRAMPC2 cells) in presence or not of 10 μM Fer-1. **C**, **D** Cytofluorimetric quantification of AnnexinV/Propidium Iodide double staining, mitochondrial superoxide production (Mitosox) and lipid peroxidation (C11-BODIPY 581/591 staining) of PCa cells treated for 48 h with 0.5 μM RSL3 or RSL3 + 100 μM FAC in the presence or not of 10 μM Fer-1. Data are the mean ± SEM of at least 3 experiments (*p < 0.05, **p < 0.01, ***p < 0.001, ^#^*p* < 0.0001).

In order to assess if iron supplementation can promote ferroptosis in PCa cell lines, we first treated for 48 h DU145 and TRAMP-C2 cells with increasing concentrations of FAC. As shown in Fig. 1B, no effect on cell proliferation was detected at the concentrations tested. In contrast, treatment with the ferroptosis inducer RSL3 resulted in a significant decrease in the number of viable cells with an IC_50_≃0.6 µM for DU145 and IC_50_≃1.2 µM for TRAMP-C2 cells (Fig. 1C). Notably, the combination treatment with RSL3 and an ineffective dose of FAC (100 µM) increased cell death and reduced the IC_50_ of RSL3 in both cell lines (IC_50_≃0.1 µM for DU145 and IC_50_≃0.4 µM for TRAMP-C2). Similarly, treatment with FAC increased the efficacy of RSL3 in an additional PCa cell line represented by the human PC3 cells (Fig. S1). Moreover, the treatment with RSL3 resulted in a significant reduction of PCa cells migration when tested in a wound healing assay and this effect was significantly increased by the addition of an ineffective concentration of FAC (100 µM) (Fig. 1D). With the purpose to better distinguish ferroptosis from other types of cell death, PCa cells were treated with RSL3 and the combination RSL3 + FAC in the presence of the selective inhibitor of the ferroptosis Ferrostatin-1 (Fer-1). As shown in Fig. 2A, Fer-1 rescued the cell death caused by RSL3 and RSL3 + FAC in both DU145 and TRAMP-C2 cells. In addition, when assayed for the impact on the clonogenic potential, treatment with RSL3 + FAC significantly reduced the number of colonies in both PCa cells and treatment with Ferrostatin-1 reverted this effect on both RSL3 and RSL3 + FAC treated cells (Fig. 2B).

Cytofluorimetric analysis of PCa cells treated with RSL3 and RSL3 + FAC revealed that the increase of cell death is paralleled by the increase of Annexin V/Propidium Iodide (PI) double positive cells, and this effect was significantly reduced by Fer-1 (Fig. 2C) [23]. Notably, the ferroptotic cell death was detected in RSL3 + FAC treated cells without the activation of the canonical apoptotic pathways as revealed by the lack of activation of the caspase 3 (Fig. S2).

Given the fundamental role of the oxidative stress and lipid peroxidation in the ferroptosis process, we investigated these events in PCa cells after their treatment with RSL3 + FAC. As shown in Fig. 2D, in both DU145 and TRAMP-C2 cells, treatment with RSL3 + FAC triggered the production of mitochondrial ROS (mtROS) as detected by Mitosox probe, and the level of lipid peroxidation detected by the BODIPY 581/591 C11 probe [24]. Notably, both the mtROS and the lipid peroxidation were significantly reduced by treatment with Fer-1 (Fig. 2D).

**Fig. 3:**
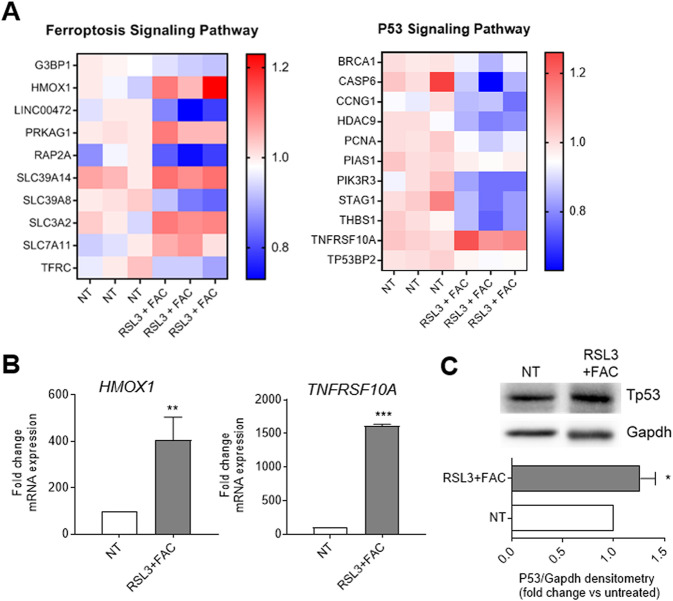
RSL3+FAC treatment impact on ferroptosis and P53 molecular pathways. **A** Heatmaps of modulated pathways showing the most differentially expressed genes in DU145 cells treated with 0.5 μM RSL3 + 100 μM FAC for 12 h. **B**, **C** The gene expression modulation of *HMOX1* and *TNFRSF10A*, respectively of the ferroptosis and P53 pathway, was confirmed by RT-qPCR analyzes and expressed as % fold change over the not treated (NT) cells (**B**), while Western blot analysis revealed an increase of Tp53 protein. Gapdh was used for normalization (**C**). Data are the mean ± SEM of at least 3 experiments (*p < 0.05, **p < 0.01, ***p < 0.001).

Finally, to deeper investigate the molecular pathways modulated by RSL3 + FAC treatment we performed a gene expression profiling (GEP) analysis on DU145 treated cells compared to untreated ones. As shown in Fig. 3A, pathway analysis of up- or down-regulated gene transcripts, performed using Ingenuity Pathway Analysis (IPA) software, revealed significant modulation of ferroptosis and p53 signaling pathways. These data were validated by RT-qPCR (Fig. 3B) and by Western blot analysis for P53 (Fig. 3C). In addition, other pathways related to intracellular stress (i.e. autophagy and protein ubiquitination) have been detected by IPA analysis confirming the activation and presence of ferroptosis in DU145 cells treated with RSL3 + FAC [25, 26] (Fig. S3).

### Iron supplementation enhances ferroptotic cell death in PCa cells in vivo

**Fig. 4:**
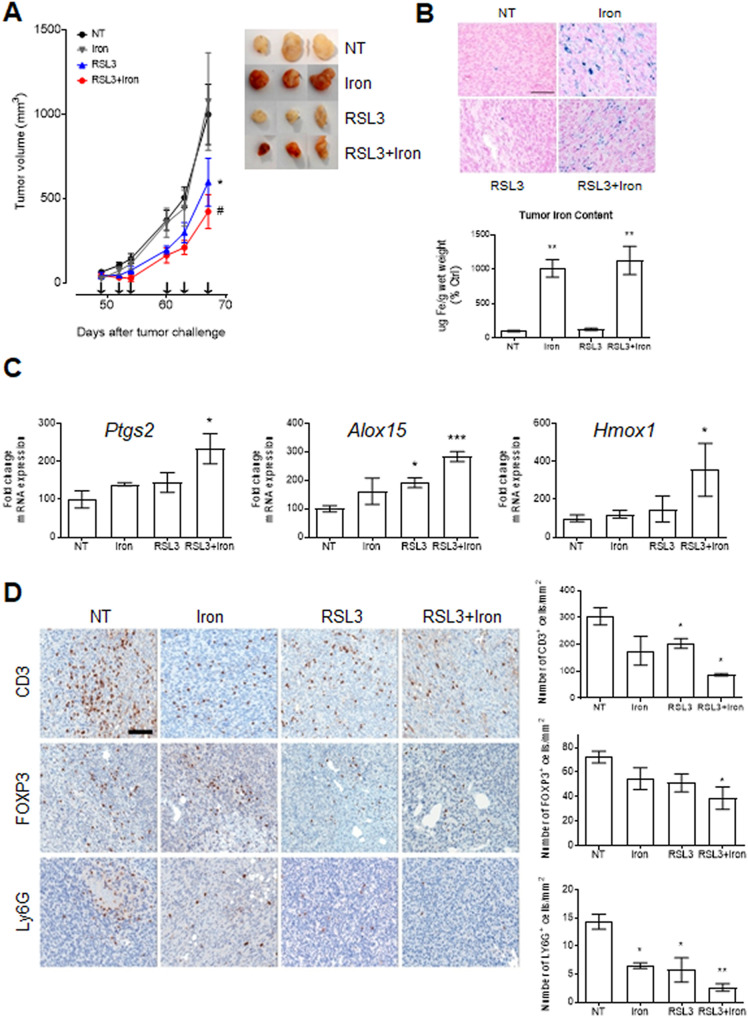
RSL3+iron impairs PCa growth in vivo. **A** In vivo growth of subcutaneous TRAMP-C2 tumors treated i.p. with iron dextran 200 mg/kg twice/week, RSL3 40 mg/kg twice/week or RSL3 + iron and compared with untreated (NT) ones. **B** Prussian Blue staining (blue dots) and total iron content assay on tumor samples. Data are expressed as % fold change over the not treated (NT). **C** qPCR of ferroptosis related genes in tumor samples. Data are expressed as % fold change over the not treated (NT). **D** Immunohistochemistry and quantification of immune populations (CD3, FOXP3 and Ly6G markers) in tumor samples expressed as positive cells/mm^2^. Data are the mean ± SEM of 8–10 tumors/group. ^*^p < 0.05, ***p < 0.001, ^#^*p* < 0.0001 refers to each group *vs* NT group. Scale bar = 100 μm.

Based on the in vitro observations, murine TRAMP-C2 cells were grafted subcutaneously into syngeneic immunocompetent C57BL/6 adult male mice. As shown in Fig. 4A, treatment with iron dextran (200 mg/kg twice a week) had no significant effect on tumor growth while treatment with RSL3 (40 mg/kg twice a week) significantly reduced tumor volume and, more importantly, the combination RSL3 + iron showed the best therapeutic profile. Of note, in any of the treatments no measurable side effects were detected as assessed by the animal’s body weight (Fig. S4A), despite the important accumulation of iron especially in the liver (Fig. S4B). Accordingly, RSL3 + iron treatment resulted in a significant reduction of tumor growth when human DU145 cells where grafted into immunodeficient NOD/Scid mice (Fig. S4C).

After surgical removal, TRAMP-C2 tumors were analyzed by qPCR and immunohistochemistry (IHC). As shown in Fig. 4B, Prussian Blue staining and the total iron content assay confirmed the presence of iron accumulation in tumor samples in iron-treated mice. In addition, a significant induction of ferroptosis-related genes (*Ptgs2*, *Alox15* and *Hmox1*) was detected in tumor samples after treatment with RSL3 + iron, thus confirming the activation of the ferroptotic process inside the tumor mass (Fig. 4C).

Finally, given the role of immunity in the tumor microenvironment, and the role of macrophages in iron accumulation [27], we took advantage of the immune-competent context of TRAMP-C2 grafts for a preliminary analysis of the immune infiltrate in treated samples. Interestingly, IHC analysis of the tumors showed a significant modulation of immune-cells in the tumor microenvironment of treated group during PCa growth (Fig. 4D). In particular, a significant reduction of CD3^+^ T lymphocytes, FOXP3^+^regulatory T cells and neutrophils (Ly6G^+^) was observed in mice treated with RSL3 + iron. In contrast, no changes in the macrophage population was observed (Fig. S4D). These observations are in line with recent findings on the possible impact of ferroptosis on the immune-modulated tumor microenvironment [28] and worth further investigation.

### Ferroptosis impairs tumor progression in the multistage TRAMP mouse model

**Fig. 5:**
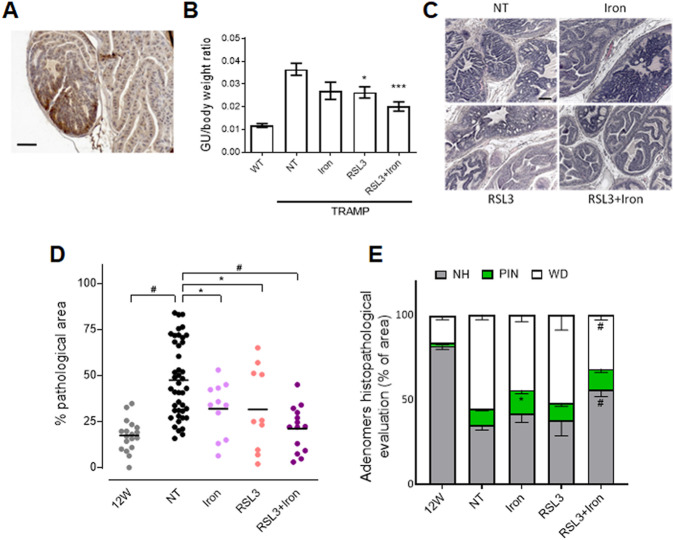
RSL3+iron impairs tumor growth in the multistage TRAMP PCa model. **A** Immunohistochemistry for transferrin receptor (TfR1) in the anterior prostate lobes of 25 week-old TRAMP mice (scale bar = 100 μm). **B** TRAMP mice were treated with iron dextran 40 mg/kg/week, RSL3 10 mg/kg/week and RSL3 + iron from 12 weeks of age until 25 weeks of age. Wild type mice, as well as no treated (NT) and treated TRAMP mice were sacrificed at 25 weeks of age and the genitourinary to body weight ratio determined. **C** Representative picture (H&E) of anterior prostate lobes of TRAMP mice at 25 weeks of age. (scale bar = 100 μm). **D** Quantification (percentage) of the pathologic area in the anterior prostate of 12 and 25-week-old untreated TRAMP (12 W and NT respectively) and treated TRAMP mice of 25 week-old. **E** Histopathologic evaluation of prostate tissue after explantation; the graph shows the percentage of normal healthy tissue (NH, gray), PIN (green), and well-differentiated tumor area (WD, white) in the prostate of untreated TRAMP (12 and 25-week-old) and treated TRAMP mice of 25 week-old. Data are the mean ± SEM of 8–10 mice/group. ^*^p < 0.05, ***p < 0.001, ^*#*^*p* < 0.0001 refers to each group vs NT group.

Given the promising results obtained in vitro and in tumor grafts in vivo, we exploited the multistage PCa tumor model represented by the TRAMP mouse, where the age-dependent transformation of the prostate starts at 8–10 weeks with intraepithelial neoplasia (PIN) and progresses to well-differentiated (WD) carcinoma [2, 19, 29]. As shown in Fig. 5A, IHC analysis on the anterior prostate lobes of 25 week-old TRAMP mice revealed a high expression of the TfR1 receptor in pathological areas compared with normal/healthy areas. This is in accordance with iron demand, typical of tumor cells [30] and suggests the possibility to overload iron in the pathological prostate context to facilitate ferroptosis triggering. This was done by treating animals with intraperitoneal injections of iron dextran (40 mg/kg once a week). The treatment with iron, RSL3 or RSL3 + iron started at 12 weeks of age, when tumor onset is evident, until 25 weeks of age. At the end of the experimental procedure the genitourinary (GU) apparatuses were removed, weighted and included for histopathological evaluation [19]. As shown in Fig. 5B, the weight of the GU apparatus was significantly reduced in mice treated with RSL3 + iron suggesting a reduction of organ oversizing/impairment due to tumor progression in this mice. Accordingly, histopathological analysis of the anterior prostate lobes revealed a significant reduction of PCa progression in animals treated with iron or RSL3 alone, and confirmed that the combination treatment was the most effective in reducing tumor burden (Fig. 5C, D). Indeed, the percentage of pathological areas was reduced from (47.5 ± 3.1)% in untreated animals to (21.2 ± 3.3)% in the RSL3 + iron treated group. In line with this therapeutic effect, the amount of normal/healthy (NH) prostate tissue in prostates treated with RSL3 + iron was increased, while well-differentiated carcinoma (WD) was reduced (Fig. 5E). Notably, in all treated groups no significant side effects were detected as assessed by the animal’s body weight (Fig. S5A) despite the accumulation of iron in the liver and spleen (Fig. S5B).

### Combined therapy of ferroptosis and androgen deprivation

Enzalutamide represents a clinically relevant second-generation androgen receptor antagonist employed for the treatment of metastatic castration-sensitive PCa endowed with significant effect in terms of prolonged survival in CRPC, although patients eventually experience disease progression [31]. Here we investigated the therapeutic potential of ferroptosis inducers combined with ADT in treating naïve PCa and prevent castrate resistant PCa settings. As shown in Fig. S6A, when DU145 and TRAMP-C2 cells were treated with combined sub-optimal doses of RSL3, FAC and enzalutamide, cell viability was significantly reduced. Also, the effect of the combination RSL3 + FAC + enzalutamide was rescued by the treatment with Fer-1, confirming the induction of ferroptotic cell death (Fig. S6A).

**Fig. 6:**
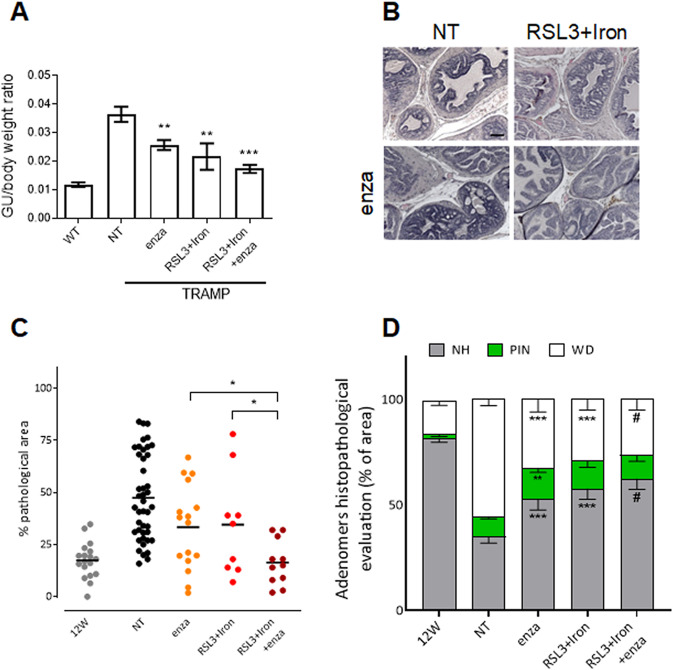
Enzalutamide potentiates the effect of RSL3+iron in the multistage TRAMP model. **A** TRAMP mice were treated with 3 mg/kg enzalutamide (enza) in combination or not with sub-optimal doses of RSL3 (5 mg/kg/week) + iron dextran (10 mg/kg/week) from 12 weeks of age until 25 weeks of age. WT, no treated TRAMP (NT) and treated TRAMP mice were sacrificed at 25 weeks of age and the genitourinary to body weight ratio determined. **B** Representative picture (H&E) of anterior prostate lobes of TRAMP mice at 25 weeks of age (scale bar = 100 μm). **C** Quantification (percentage) of the pathologic area in the anterior prostate of 12 and 25-week-old untreated TRAMP (12 W and NT respectively) and treated TRAMP mice of 25 week-old. **D** Histopathologic evaluation of prostate tissue after explantation; the graph shows the percentage of normal healthy tissue (NH, gray), PIN (green), and well-differentiated tumor area (WD, white) in the prostate of untreated TRAMP (12 and 25-week-old) and treated TRAMP mice of 25 week-old. Data are the mean ± SEM of 8–10 mice/group. ^*^p < 0.05, ***p < 0.001, ^*#*^*p* < 0.0001 refers to each group *vs* NT group if not differently specified.

In vivo, TRAMP mice were treated with the combination of the three drugs (RSL3 + iron+enzalutamide) from 12 to 25 weeks of age to assess the efficacy of this treatment. In this case, lower doses of RSL3 (5 mg/kg) and iron dextran (10 mg/kg) were used if compared with previous experiments (RSL3 10 mg/kg and iron dextran 40 mg/kg; see Fig. 5). In accordance with in vitro data, the weight of the GU apparatus (Fig. 6A) and the histopathological analysis of the anterior prostate lobes (Fig. 6B, C) indicate that the triple combination exerted a superior anti-tumor effect if compared with untreated/control group and with mice treated with enzalutamide alone or with the RSL3 + iron combination. The percentage of pathological areas was reduced from (47.5 ± 3.1)% in control animals to (16.4 ± 4.9)% in RSL3 + iron+enzalutamide treated mice, thus maintaining levels of transformed adenomers similar to the initial stage at 12 weeks. Indeed, the percentage of normal/healthy prostate tissue increased to (62.3 ± 5)% in the triple combination-treated group (Fig. 6D). Notably, no measurable side effects were detected as assessed by animal body weight (Fig. S6B) despite the important accumulation of iron in the liver and in the spleen (Fig. S6C) that resulted slightly reduced if compared with higher doses treatments used in previous experiments (Fig. S5B).

## Discussion

Ferroptosis is a recently described form of cell death triggered by abundant cytosolic labile iron that promotes membrane lipid peroxidation. Susceptibility to ferroptosis has been reported in different cancer types like renal cell carcinoma [32], non‐small cell lung cancer [33], breast cancer [34] and pancreatic cancer [35], where it causes tumor cells death. Indeed, it has been reported that tumor cells act in order to avoid and escape ferroptosis, in fact inactivation of the oncosuppressor P53 pathway during the onset of most human cancers was also associated with ferroptosis suppression [36]. Moreover, it has been reported that melanoma cells tend to avoid ferroptosis metastasizing through the lymphatic system, where the concentration of GSH and oleic acid are higher and free iron is less present, instead of the blood circulation [37]. Similarly, it has been observed that lung adenocarcinomas preferentially select expression pathways that confers resistance to high oxygen tension and protects cancer cells from ferroptosis triggered by oxidative damage [38].

In PCa, it has been reported that iron accumulation triggers cell death in vitro and may strengthens the efficacy of anti-androgen treatment in vivo in tumor graft models [21]. In addition, the potential to exploit ferroptosis inducers like RSL3 and Erastin in PCa has been recently reported by Goochani and colleagues using human PCa cells in vitro and in vivo xenografts in immunocompromised mice [22].

Our data demonstrated that in vitro iron supplementation strongly potentiates the RSL3 effect in compromising the proliferative, clonogenic and migratory capacity of human and murine PCa cells. Notably, the combined treatment with RSL3 and iron significantly increased the intracellular oxidative stress resulting in huge lipid peroxidation, without the activation of canonical apoptotic pathways. These observations were confirmed in vivo, where combined treatment significantly impaired the growth of human (DU145) and murine (TRAMP-C2) xenografts, and this was accompanied by iron accumulation and ferroptosis activation in tumor samples. The efficacy of iron supplementation in combination with RSL3 treatment was further confirmed in the multistage murine TRAMP model that represents a more reliable and close to the clinic model mimicking PCa onset and progression. It is worth to mention that in the syngeneic/immunocompetent PCa model a significant change in the tumor-infiltrating immune populations was observed, with a reduction of CD3^+^ and FOXP3^+^ T lymphocytes, as well as of neutrophils. Notably, it has been recently described that a major immune-suppressive population, the neutrophils (PMNs) myeloid-derived suppressor cells (PMN-MDSCs), spontaneously die in the tumor microenvironment by ferroptosis [28]. In addition, ferroptosis limits the activity of T cells through the release of oxygenated lipids [28]. Thus, even if out of the scope of the current work, a deeper investigation on the interplay between ferroptosis and the tumor immune milieu would be required to better understand the role and to properly exploit ferroptosis as a potential mechanism to rewire the immunosuppressive mechanism during tumor growth.

Finally, we validated the superior therapeutic potential of iron supplementation and RSL3 when combined with ADT in vitro and in the multistage TRAMP mouse model. Indeed, treatment with enzalutamide significantly enhanced the anti-tumor efficacy of iron and RSL3 also at sub-optimal doses. Notably, the triple treatment maintained tumor dimension at initial/basal levels significantly improving the efficacy of enzalutamide and possibly preventing (or delaying) the onset of the enzalutamide-resistant state.

Altogether this work shows for the first time that iron supplementation may be used to enhance ferroptosis adding a new piece to the puzzle to better understand and exploit this iron-mediated cell death mechanism in PCa therapy. Moreover, under a translational point of view the possibility to delay or prevent the onset of the CRPC represents a promising and key aspect for this critical clinical condition with poor perspectives and no optional treatment.

## Materials and methods

### Reagents and cell cultures

TRAMP-C2 cells (representing a murine prostate androgen-dependent adenocarcinoma; ATCC number CRL-2731) are cultured as described in [19]. DU145 (ATCC HTB-81) and PC3 (ATCC CRL-1435) are human prostate cancer cells cultured in RPMI 1640 with 10% FBS. Cells were kept at low passage, and periodically assayed for negativity to Mycoplasma. RSL3 (#SML2234), Ferric Ammonium Citrate (FAC, #F5879) and Ferrostatin-1 (#SML0583) were provided by Sigma-Aldrich. Enzalutamide (MDV3100) was provided by Selleckchem.

### Cell proliferation assays

DU-145 and TRAMP-C2 cells were seeded in 48-well plates at 15,000 and 8000 cells/cm^2^ respectively in complete growth medium. The day after cells were treated with increasing concentrations of FAC (25–500 μM), RSL3 (0.0001–10 μM) and with the combination of these two drugs (0.0001–10 μM RSL3 + 100 μM FAC) in presence or not of 10 μM Ferrostatin-1.

For combination studies with androgen deprivation therapy, cells were treated with 5 μM enzalutamide in combination or not with sub-optimal doses of RSL3 + FAC. In particular, DU145 cells were treated with 0.010 μM RSL3 + 50 μM FAC while TRAMP-C2 cells with 0.001 μM RSL3 + 50 μM FAC. For both studies, after 48 h of incubation cells were detached, stained with Propidium iodide (PI) and viable cell counting were performed using the counting function of the MACSQuant® Analyzer (MiltenyiBiotec).

### Wound healing assay

Confluent cells monolayers were wounded using a 200 μL tip, then cells were maintained in complete medium and treated with 100 μM FAC and RSL3 (0.3 μM for DU-145 cells and 1.0 μM for TRAMPC2 cells). After 24 h, the wounds were photographed under an inverted Zeiss Axiovert 200 M photomicroscope and the migration was quantified by computer assisted image analysis.

### Clonogenic assay

Cells were plated 50 cells/cm^2^ in 6-well plates in complete growth medium and after 24 h were treated with 1 μM FAC and RSL3 (0.1 μM for DU-145 cells and 0.5 μM for TRAMPC2 cells) in presence or not of 10 μM Ferrostatin-1. Colonies of cells were allowed to grow for one week, then stained using a 0.1% crystal violet/20% methanol solution. Plates containing colonies were photographed and cell colonies quantified by computerized image analysis.

### Cytofluorimetric analyses

Cytofluorimetric analyses were performed using the MACSQuant Analyzer® (MiltenyiBiotec). Mitochondrial superoxide production and lipid peroxidation levels were determined using specific fluorescent probes MitoSox and C11-BODIPY 581/591 (ThermoFischer Scientific), respectively. Apoptotic cell death was assessed by Annexin-V/PI double staining Immunostep and activated caspase-3 levels were determined using the antibody against cleaved caspase-3 (#9661 Cell Signaling).

### RT-qPCR analysis

TRIzol Reagent (Invitrogen) was used for RNA extraction and followed by treatment with DNAse. Two μg of total RNA were retro-transcribed with MMLV reverse transcriptase (Invitrogen) using random hexaprimers. Then, cDNA was analyzed by Real time PCR using specific primers indicated in Supplementary Table 1.

### Genome-wide expression profiling (GEP)

Total RNA of DU145 cells treated for 12 h with 100 μM FAC, 0.5 μM RSL3 or RSL3 + FAC was extracted and processed as in [39]. After quality controls, Analysis of Variance (ANOVA) test was performed to assess the effects of RSL3 + FAC treatment on gene expression, comparing DU145 cells that received the combined therapy vs untreated ones (NT). *P* value < 0.05 and fold change ±1.2 was used to select differentially expressed genes that were imported into the Ingenuity Pathways Analysis (IPA) software (Ingenuity Systems). IPA through precise algorithms identified significant networks, top functions and canonical pathways associated with the differentially expressed genes.

### Western blot analysis

PCa cells were treated for 24 h with 100 μM FAC, 0.5 μM RSL3 or RSL3 + FAC and homogenized in NP-40 lysis buffer (1% NP-40, 20 mM TrisHCl pH 8, 137 mM NaCl, 10% glycerol, 2 mM EDTA, 1 mM sodium orthovanadate, 10 g/mL aprotinin, 10 g/mL leupeptin). Protein concentration in the supernatants was determined using the Bradford protein assay (Bio-Rad Laboratories). Then, 60 μg protein/sample were loaded in SDS-PAGE and analyzed by WB using specific primary antibodies against Transferrin Receptor (TfR1) (#13-6800Invitrogen), Ferritin (Ft) (#F5012 Sigma), Tubulin (#T5168 Sigma), Tp53 (#2524Cell Signaling) and Gapdh (#sc-25778 Santa Cruz Biotechnology).

### Histochemistry

Prussian Blue staining was performed on histological section permeabilized with PBS/Triton 0.5% and then incubated for 1 h with 4% potassium ferrocyanide in 6% hydrochloric acid. Images of the staining was acquired with Zeiss Axiovert 200 M.

### Immunohistochemistry

Samples were fixed and processed as described in [19]. The following primary antibodies were used: mouse monoclonal anti-CD3 (clone SP7, 1:70, Leica, #565-LCE) and anti-FOXP3 (1:200, Abcam, ab22510), rat monoclonal anti-Ly6G (clone 1A8, 1:400, Cederlane, #ABF118UD), rabbit polyclonal anti-IBA1 (1:300, Wako, #019-19741), and mouse monoclonal anti-TfR (1:800 #M36008 Invitrogen). Detection was done using EnVision+ System-HRP Labeled Polymer anti-mouse or anti-rabbit (Dako) or using Rat-on-Mouse HRP-Polymer (Biocare Medical) followed by DAB as chromogen. Hematoxylin counterstaining was performed on all sections.

Positive cells count was carried out with Aperio Imagescope on digitalized sections acquired using the Aperio CS2 digital scanner and ScanScope software. The analysis was perform using IHC nuclear Image Analysis and Positive Pixel Count v9 9.0 Algorithm (Imagescope, Leica Biosystem). The whole tumor area was considered for the analysis and necrotic areas were excluded.

### Evaluation of iron content

Photometric total iron content evaluation was done as described in [40] using 50 mg of tumor, liver or spleen tissue digested in an acidic solution and processed with working chromogen reagent. Iron concentration was determined measuring the absorbance at 535 nm.

### In vivo experiments

Animal experiments were authorized by the local animal ethics committee (OPBA, University of Brescia) and were performed in accordance with national guidelines and regulations.

#### Heterotopic tumor models

Twelve-week-old C57BL/6 J or NOD/Scid male mice were injected subcutaneously with 10 × 10^6^ TRAMP-C2 or 5 × 10^6^ DU145 cells, respectively. When tumors were palpable (~80 mm^3^), mice were treated intraperitoneally (i.p.) with iron dextran 200 mg/kg twice/week (#D8517 Sigma), RSL3 (dissolved in DMSO) 40 mg/kg twice/week or RSL3 + iron. Mice in the control group (NT group) were treated with vehicle (DMSO). Tumors were measured with a caliper and volume calculated as described in [41]. At the end of the experimental procedure tumors were removed, weighted and processed for further analyses. Liver and spleen were collected and processed for bathophenanthroline assay.

#### TRAMP mouse model

TRAMP mice (C57BL/6-Tg(TRAMP)8247Ng/J) [42] were obtained from The Jackson Laboratory (Bar Harbor, ME, USA) and bred as described in [19]. Twelve-week-old mice were treated i.p. with iron dextran 40 mg/kg/week, RSL3 (dissolved in DMSO) 10 mg/kg/week or RSL3 + iron starting at 12 week of age until 25 week of age.

For combination studies with androgen deprivation therapy, TRAMP mice were treated with enzalutamide (3 mg/kg) in the drinking water from 12 week of age until 25 week of age in combination or not with sub-optimal doses of RSL3 + iron dextran (5 mg/kg/week RSL3, 10 mg/kg/week iron dextran). At week 25 mice were sacrificed and prostates processed as described in [20, 43].

### Murine prostate histopathological analysis

The genitourinary apparatus was explanted and FFPE then anterior prostate sections of 7 μm were stained with hematoxylin and eosin (H&E) and histological features were evaluated. The whole section of the anterior prostate was acquired at ×20 magnification with the “Mosaic Tool” at the Zeiss Axiovert 200 M microscope (Carl Zeiss, Italy). The quantification of the pathological areas was performed with the AxioVision LE64 software.

### Statistical analyses

Student’s *t* test for unpaired data (2-tailed) was used, and Prism 5 (GraphPad Software) as software to test the probability of significant differences between two groups of samples. For tumor volume statistics a 2-way analysis of variance, and Bonferroni correction was used.

## Supplementary information

Original Data File

Supplementary material

## Data Availability

Further information and requests for reagents and resources should be directed to and will be made available by the corresponding authors upon reasonable request.
