# Supplementary figures and images for: Iron supplementation enhances RSL3-induced ferroptosis to treat naïve and prevent castration-resistant prostate cancer
Source: Cell Death Discov. 2023 Mar 6;9:81. doi: 10.1038/s41420-023-01383-4 (PMC9986230; doi:10.1038/s41420-023-01383-4)

**FIGURE 1**

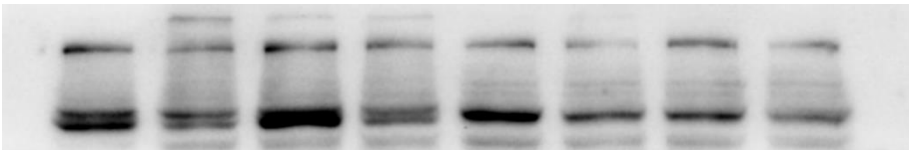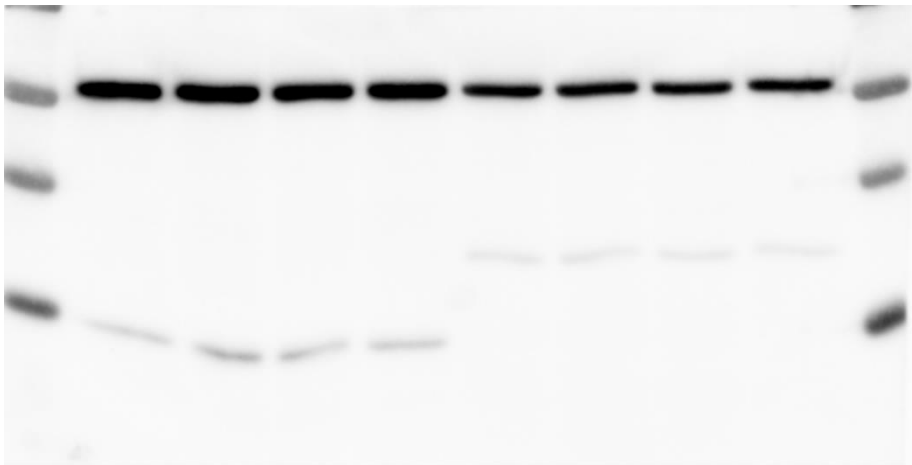

**FIGURE 1**

**DU145cells**

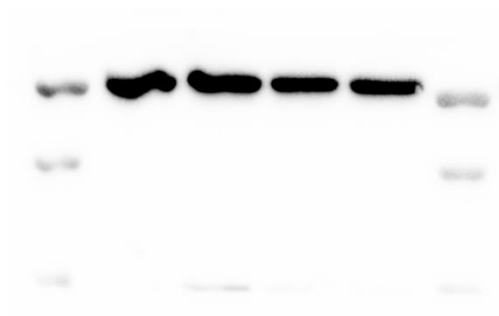

Tubulin

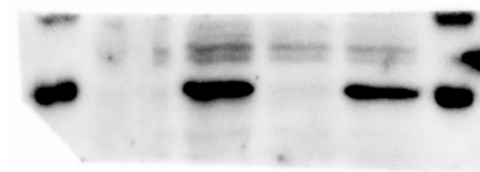

Ferritin

**TRAMP-C2**

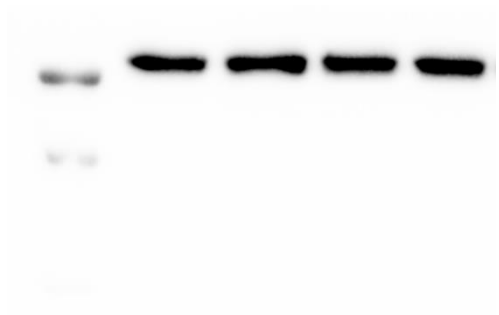

Tubulin

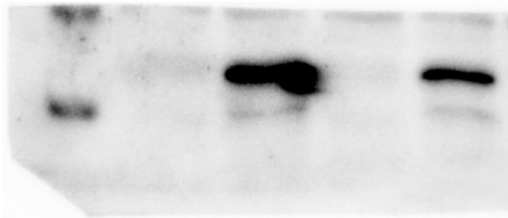

Ferritin

FIGURE 3

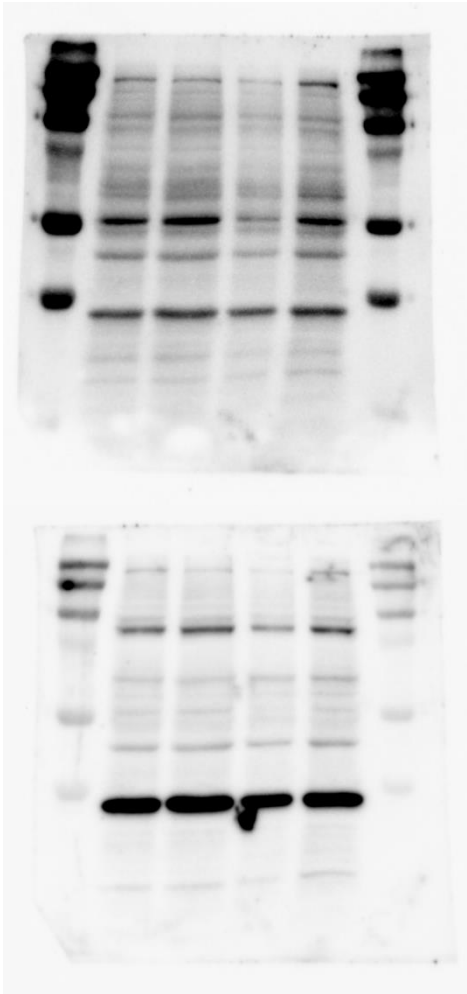

Tp53

Gapdh

Supplement: Supplementary file 1 — Original Data File [file 41420_2023_1383_MOESM1_ESM.pdf]
