# Supplementary material for: Iron supplementation enhances RSL3-induced ferroptosis to treat naïve and prevent castration-resistant prostate cancer
Source: Cell Death Discov. 2023 Mar 6;9:81. doi: 10.1038/s41420-023-01383-4 (PMC9986230; doi:10.1038/s41420-023-01383-4)
Supplement: Supplementary file 2 — Supplementary material [file 41420_2023_1383_MOESM2_ESM.pdf]

SUPPLEMENTARY FIGURES

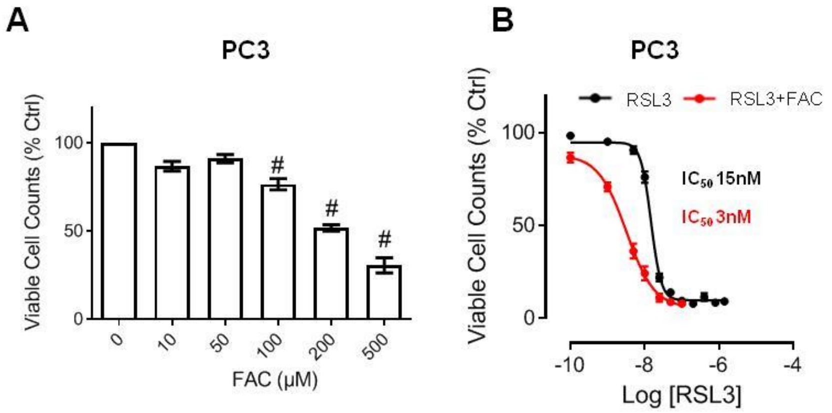

Figure S1. *In vitro* effect of RSL3+FAC treatment on PC3 cells. Viable cell count of PC3 cells treated for 48 h with increasing concentrations of FAC (10-500 µM) (**A**), or RSL3 (0.0001-1 µM) and RSL3+FAC (0.0001-0.1 µM RSL3+10 µM FAC) (**B**). Data are the mean ± SEM of at least 3 experiments, $^{\#}p < 0.0001$.

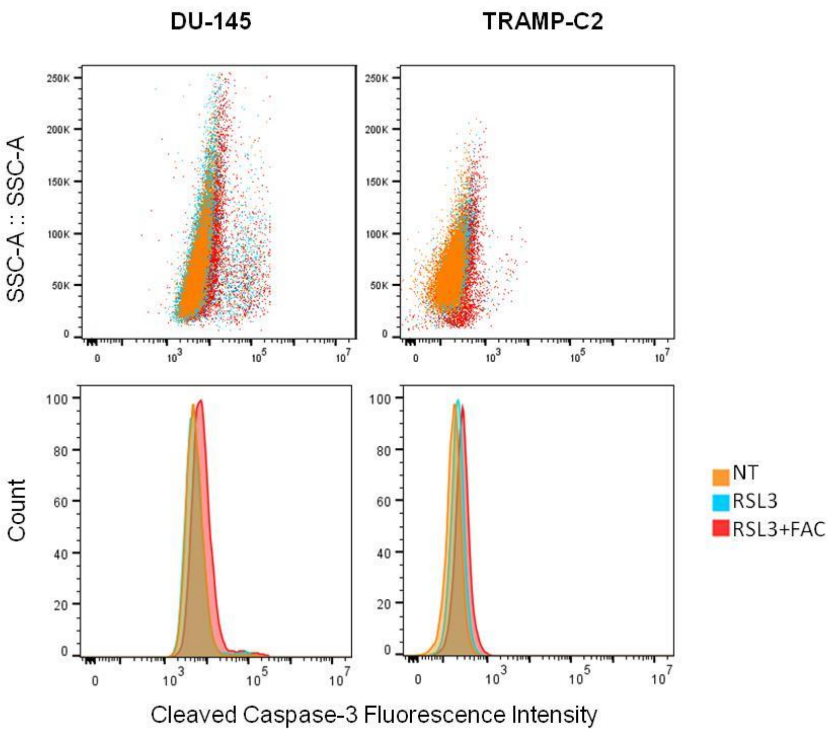

Figure S2. RSL3+FAC treatment does not activate Caspase 3 in PCa cells. Flow cytometry analysis for cleaved Caspase-3 activity in DU145 and TRAMP-C2 cells treated with 0.5μM RSL3 or RSL3+100μM FAC and compared to untreated samples. Data are the mean ± SEM of at least 3 experiments.

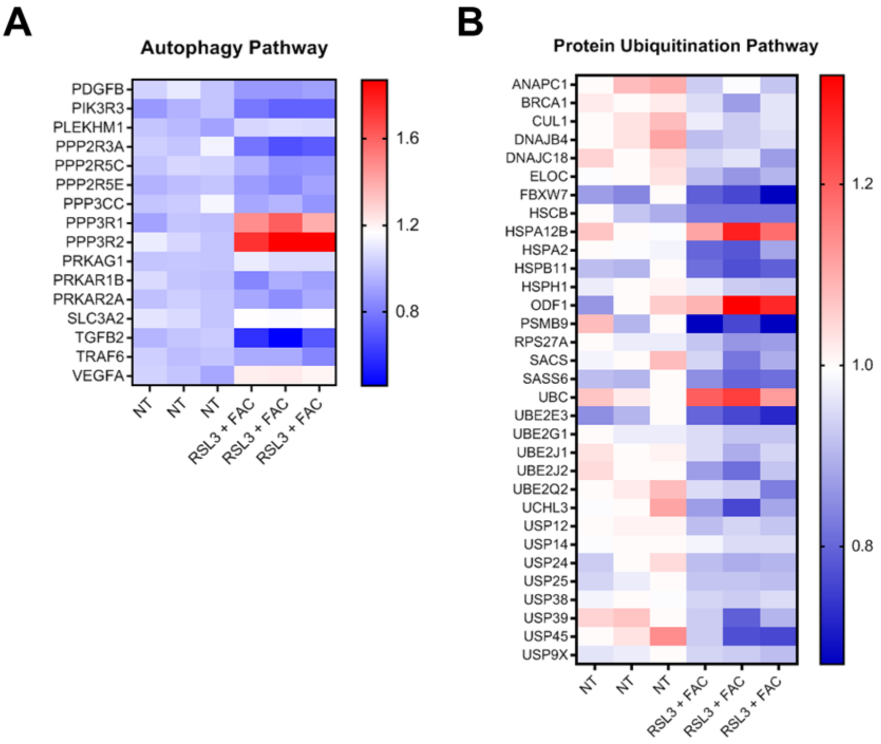

Figure S3. Oxidative stress pathways modulated by ferroptosis. DU145 cells were treated with 0.5µM RSL3+100µM FAC for 12 hours and heatmaps of the most differentially expressed genes in two pathways related to intracellular stress (autophagy (**A**) and protein ubiquitination (**B**) pathway) were generated by IPA software.

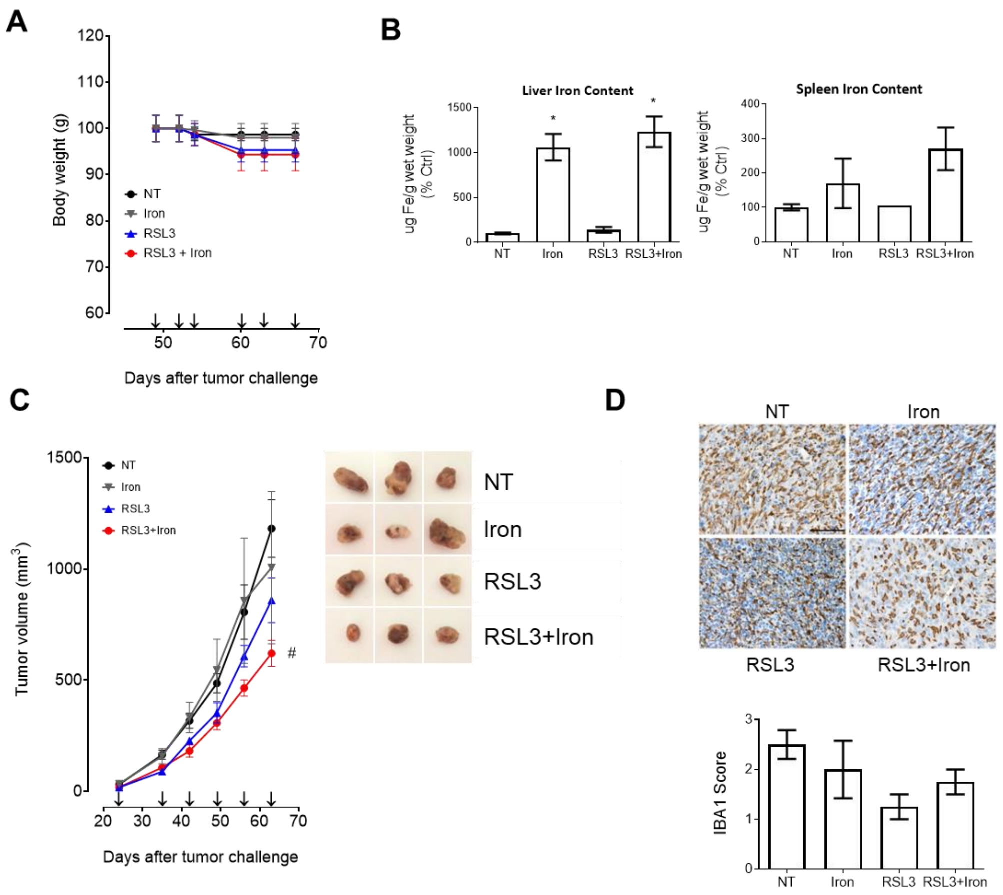

Figure S4. ***In vivo*** **effect of RSL3+FAC treatment on PCa cells.** For tumor subcutaneous grafts experiments performed with TRAMP-C2 cells (in Figure 4), animal weight was measured over the treatment and plotted (**A**), and iron accumulation in the liver and spleen was shown by a photometric iron assay (**B**). Data are the mean ± SEM of 8-10 mice/group (*$p < 0.05$). **C)** *In vivo* growth of subcutaneous DU145 tumors treated i.p. with iron dextran 200mg/kg twice/week, RSL3 40mg/kg twice/week or RSL3+iron and compared with untreated ones. Data are the mean ± SEM of 8-10 tumors/group ($^{\#}p < 0.0001$). **D)** The decreased expression of IBA1 in TRAMP-C2 subcutaneous grafts treated with RSL3+iron was detected by immunohistochemistry and quantified by scoring the immunostaining. Data are the mean ± SEM of 8-10 tumors/group.

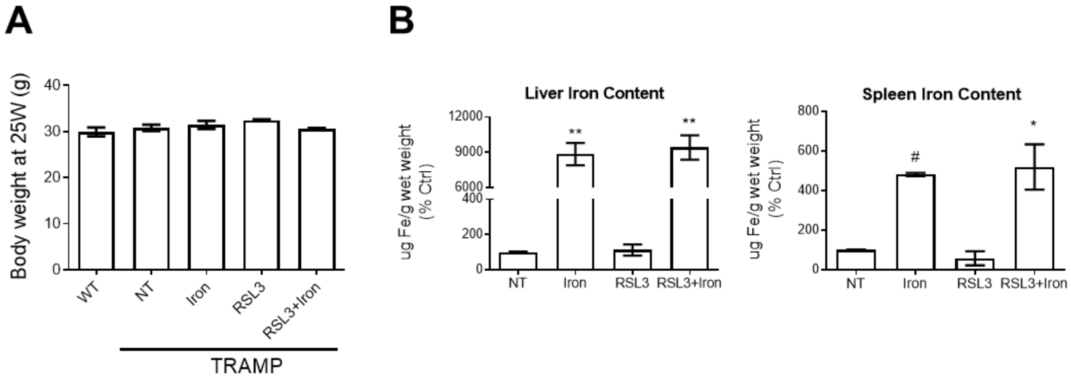

Figure S5. RSL3+iron treatment in the multistage TRAMP PCa model. A) Wild type C57BL/6J mice, as well as no treated (NT) and treated TRAMP mice were weighted at the end of the treatment (25 weeks of age) to confirm the absence of significant side effects. **B)** Iron accumulation in the liver and spleen was shown by a photometric iron assay. Data are the mean ± SEM of 8-10 mice/group ($^{*}p < 0.05$, $^{**}p < 0.01$, $^{\#}p < 0.0001$).

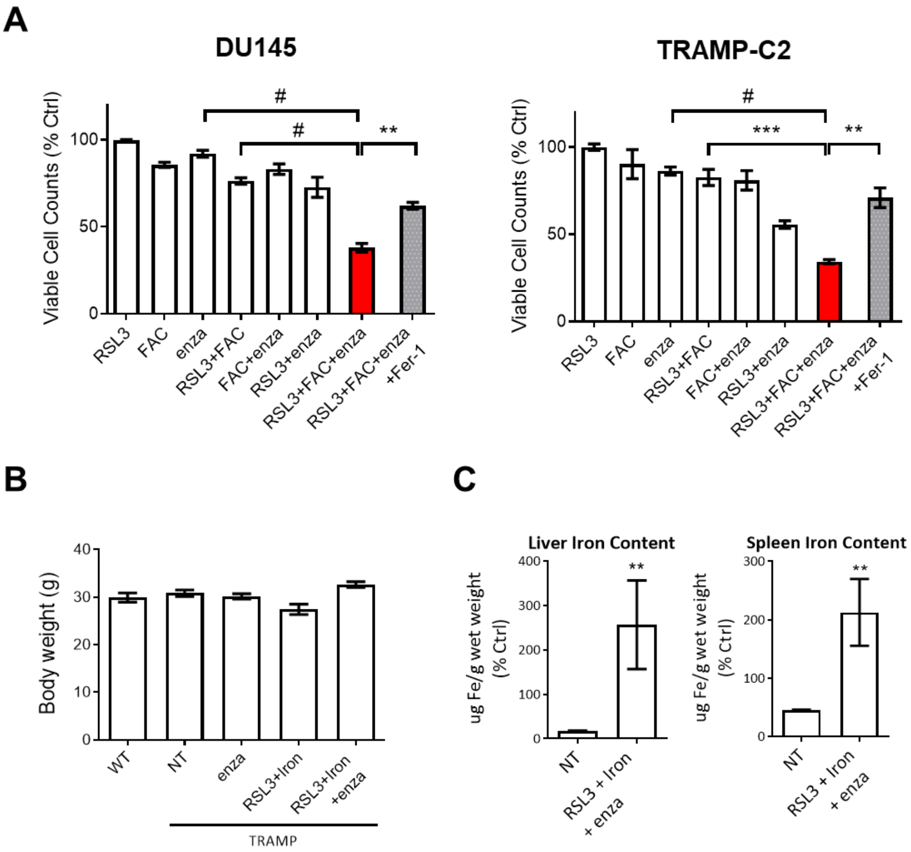

Figure S6. Enzalutamide potentiates the effect of RSL3+iron in PCa cells. A) Viable cell count of DU145 and TRAMP-C2 cells treated for 48 h with 5μM enzalutamide in combination or not with sub-optimal doses of RSL3 (0.010μM for DU-145 cells and 0.001μM for TRAMPC2 cells) and 50μM FAC. **B**) TRAMP mice were treated with 3mg/kg enzalutamide (enza) in combination or not with sub-optimal doses of RSL3+iron dextran (5mg/kg/week RSL3, 10mg/kg/week iron dextran). Mice were weighted at the end of the treatment to confirm the absence of significant side effects. **C**) Photometric iron evaluation showed iron accumulation in the liver and the spleen of iron treated mice. Data are the mean ± SEM of 8-10 mice/group (*$**p < 0.01$, $^{\#}p < 0.0001$).*

Table 1. Primers sequence used in the study.

GENE		PRIMER SEQUENCE (5' > 3')
Hs_HMOX1	For	AAGACTGCGTTCCTGCTCAAC
	Rev	AAAGCCCTACAGCAACTGTCG
Hs_TNFRSF10A	For	TGACTTTGGTTGTTCCGTTG
	Rev	TCTCGTTGTGAGCATTGTCC
Hs_GAPDH	For	GAAGGTCGGAGTCAACGGATT
	Rev	TGACGGTGCCATGGAATTTG
Mm_Ptgs2	For	ATTCTTTGCCCAGCACTTCA
	Rev	GGGATACACCTCTCCACCAA
Mm_Alox15	For	TTCACAGGTTCTGGGGACA
	Rev	GGCAGGGAGACAAGTAGACC
Mm_Hmox1	For	AAGCCGAGAATGCTGAGTTCA
	Rev	GCCGTGTAGATATGGTACAAGGA
Mm_Gapdh	For	CCCTTAAGAGGGATGCTGCC
	Rev	TACGGCCAAATCCGTTCACA

SUPPLEMENTARY METHODS

Histochemistry.. Prussian Blue staining was performed to identify iron. Briefly, brain sections were washed twice with PBS, permeabilized with PBS/Triton 0.5% and then incubated for 1 h with 4% potassium ferrocyanide in 6% hydrochloric acid. Prussian Blue staining was acquired with Zeiss Axiovert 200M.

Immunohistochemistry. Samples were fixed overnight in 10% formalin (05-01004F BioOptica), dehydrated in a graded ethanol series, embedded in paraffin and cut. Four-µm sections were deparaffinized with xylene, re-hydrated and incubated with 3% H_2O_2 in methanol for 20 min to inhibit endogenous peroxidase activity. Immunostaining was performed upon microwave or thermostat bath oven epitope retrieval in ethylene diamine tetra-acetic acid (EDTA) buffer (pH 8.00). The following primary antibodies were used: mouse monoclonal anti-CD3 (clone SP7, 1:70, Leica, #565-LCE) and anti-FOXP3 (1:200, Abcam, ab22510), rat monoclonal anti-Ly6G (clone 1A8, 1:400, Cederlane, #ABF118UD), rabbit polyclonal anti-IBA1 (1:300, Wako, #019-19741), and mouse monoclonal anti-TfR(1:800 #M36008 Invitrogen). Immunoreaction was revealed by using EnVision+ System-HRP Labelled Polymer anti-mouse or anti-rabbit (Dako) or using Rat-on-Mouse HRP-Polymer (Biocare Medical) followed by DAB as chromogen: sections were counterstained with hematoxylin.
Positive cells count was performed using Aperio Imagescope on digitalized sections. Briefly, stained slides were acquired using the Aperio CS2 digital scanner and ScanScope software (Leica biosystems, Wetzlar, Germany). The analysis was perform using IHC nuclear Image Analysis and Positive Pixel Count v9 9.0 Algorithm (Imagescope, Leica Biosystem). The whole tumor area was considered for the analysis, with the exclusion of necrotic areas. Data are expressed as absolute number of $CD8^+$ cells per mm^2.

Iron assay. Photometric total iron content evaluation was done as described in [24]. Briefly, 50mg of tumor, liver or spleen tissue were digested in an acidic solution (3M HCl and 0.6M trichloroacetic acid) at 65°C for 16 h. Then, samples were clarified by centrifugation and 2µL of the supernatant were added to 200µL of working chromogen reagent (0.01% bathophenanthroline and 0.1% thioglycolic acid in 45% sodium acetate). The solutions were then incubated for 30 min at room temperature until color development and the absorbance measured at 535 nm.
